# 

**Published:** 1896-01

**Authors:** 


NOTICE.—Our offer of a thermometer to *all* cash-in-advance subscribers is withdrawn. *New* subscribers, paying full year in advance, may still get the thermometer. Book offer still open to all. "Antisepsis and Antiseptics," "Stories of a Country Doctor."

